# Evolutionary Conservation Levels of Subunits of Histone-Modifying Protein Complexes in Fungi

**DOI:** 10.1155/2009/379317

**Published:** 2009-05-18

**Authors:** Hiromi Nishida

**Affiliations:** Agricultural Bioinformatics Research Unit, Graduate School of Agricultural and Life Sciences, The University of Tokyo, 1-1-1 Yayoi, Bunkyo-ku, Tokyo 113-8657, Japan

## Abstract

Eukaryotes possess a variety of histone-modifying protein complexes. Generally, a histone-modifying protein complex consists of multiple subunits, that is, a catalytic subunit and the associated subunits. In this study, I analyzed 62 and 48 subunits of the histone-modifying protein complexes of *Saccharomyces cerevisiae* and *Schizosaccharomyces pombe*, respectively. The evolutionary conservation levels of the 110 subunits were measured. The measurements revealed that the conservation levels of the catalytic subunits are significantly higher than those of the associated subunits of the histone acetyltransferase and deacetylase complexes; however, the conservation level of the catalytic subunits is similar to that of the associated subunits of the histone methyltransferase complexes. Thus, in the fungal histone acetylation and deacetylation systems, the catalytic subunits of histone-modifying protein complexes are conserved and the associated subunits are evolutionary lineage-specific. In contrast, in the fungal histone methylation system, both the catalytic and the associated subunits are evolutionary lineage-specific.

## 1. Introduction

Chromatin is the most important structure for the maintenance of the eukaryotic genomic DNA. The eukaryotic genomic DNA is packaged with histone proteins to form nucleosomes (the fundamental repeating unit of chromatin). Chromatin structure depends on the modification of nucelosome core histones [1, 2]. Generally the proteins that are evolutionarily conserved and distributed among a wide range of organisms play an important role in the biological processes [3]. Eukaryotes possess a variety of histone-modifying protein complexes [4, 5]. Some subunits of histone-modifying protein complexes have been evolutionarily conserved among eukaryotes, while some subunits are evolutionary lineage-specific. For example, the histone-modifying protein Clr4 methylates histone H3 at lysine 9, which plays a major role in RNA-mediated heterochromatin formation in the fission yeast *S. pombe* [5]. However, the budding yeast *Sacchromyces cerevisiae* lacks Clr4 homologue [6]. There has been considerable progress in the studies on fungal histone modifications in *S. cerevisiae* and *S. pombe*. Most gene functions in other fungi have been annotated based on the structural similarity of their genes with the genes of the abovementioned 2 yeasts whose functions have been studied well (inferred from the results of biological experiments). In this study, I used the subunits of histone-modifying protein complexes extracted from *Saccharomyces* and *Schizosaccharomyces*. The purpose of this study is to show the evolutionary conservation levels of the subunits of fungal histone-modifying protein complexes.

## 2. Materials and Methods

The sequences of coding for subunits of histone-modifying protein complexes in *Saccharomyces* and *Schizosaccharomyces* were extracted from 2 major genome databases, *Saccharomyces* Genome Database (http://www.yeastgenome.org/), and *S. pombe* GeneDB (http://www.genedb.org/genedb/pombe/) at the Wellcome Trust Sanger Institute. In order to identify proteins homologous to the extracted *Saccharomyces* and *Schizosaccharomyces* proteins, a BLASTP search was performed for 9 complete fungal genomes (6 ascomycetes species, namely, *Aspergillus fumigatus, Kluyveromyces lactis, Neurospora crassa, S. cerevisiae, S. pombe*, and *Yarrowia lipolytica*; 2 basidiomycetes species, namely, *Cryptococcus neoformans, Ustilago maydis*, and 1 microsporidium, namely, *Encephalitozoon cuniculi*) in the Kyoto Encyclopedia of Genes and Genomes (KEGG) database [7]. Based on the *E* values of the BLASTP search results, I classified 6 evolutionary conservation levels and scored them as follows: score 0, not detected; score 1, *E* value > 10^0^; score 2, 10^−50^ < *E* value ≤ 10^0^; score 3, 10^−100^ < *E* value ≤ 10^−50^; score 4, 10^−150^ < *E* value ≤ 10^−100^; score 5, *E* value ≤ 10^−150^. Based on the total of their scores, the subunits were ranked.

The Molecular Evolutionary Genetics Analysis (MEGA) software [8] was used to generate a neighbor-joining tree with 1000 bootstrap replicates from multiple alignments with all the gap sites deleted. A total of 496 amino acid sites were considered. PHYLIP software [9] was used to generate a maximum likelihood tree with 100 bootstrap replicates. The JTT model was used as the model of amino acid substitution. Number of times to jumble in the PROML program was 2. 

## 3. Results and Discussion

From the 2-yeast genome databases, I extracted 62 and 48 subunits of histone-modifying protein complexes of *S. cerevisiae* and *S. pombe*, respectively. Among the 110 subunits, 34 *Saccharomyces* and 24 *Schizosaccharomyces* proteins were catalytic subunits; the others were associated complex subunits. The evolutionary conservation levels of the 62 *Saccharomyces* and 48 *Schizosaccharomyces* proteins are shown in Tables 1 and 2, respectively. 

The histone acetyltransferase (HAT) catalytic subunit ELP3 was found to be the most conserved among the 62 *Saccharomyces* and 48 *Schizosaccharomyces* subunits. The main acetylation sites of ELP3 are lysine-14 of histone H3 and lysine-8 of histone H4 [10]. In addition, ELP3 is an integral subunit of elongating RNA polymerase II holoenzyme in *S. cerevisiae*, which is involved in transcription-associated chromatin modification and remodeling [11, 12]. The deletion of *ELP3* gene in yeast confers slow growth adaptation, slow gene activation, and temperature sensitivity [11]. The ELP3 protein's function may be so important for fungi (eukaryotes) that it is the most conserved.

**Figure 1:**
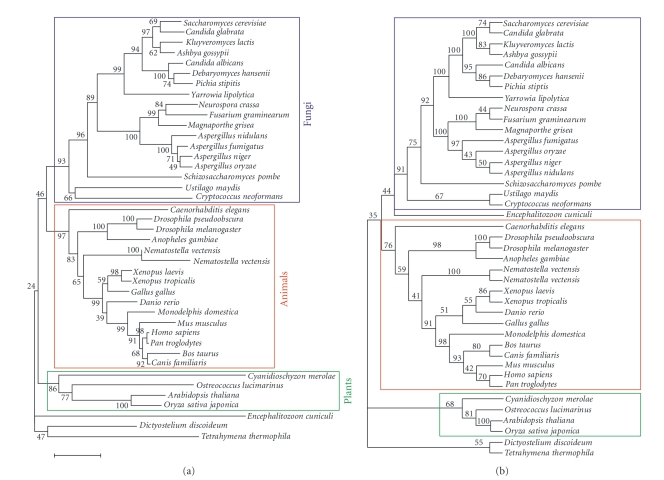
Phylogenetic relationships among the histone acetyltransferase catalytic subunit ELP3 and its homologues. A total of 496 amino acid sites were considered from multiple alignments with all the gap sites deleted. (a) Neighbor-joining tree was generated with 1000 bootstrap replicates using the MEGA software [8]. The number at each node represents the percentage obtained in the bootstrap analysis. The bar indicates a 5% difference in the evolutionary distance. (b) Maximum likelihood tree was generated with 100 bootstrap replicates using the PHYLIP software [9]. The number at each node represents the percentage obtained in the bootstrap analysis. The JTT model was used as the model of amino acid substitution. Number of times to jumble in the PROML program was 2.

The phylogenetic tree based on ELP3 and its homologues show that these proteins are present across eukaryotes (Figures 1(a) and 1(b)). The phylogenetic relationships among fungal ELP3 and its homologues are consistent with the fungal classification [13]. Interestingly, the microsporidium *E. cuniculi* is not included in the fungal lineage in the neighbor-joining tree (Figure 1(a)), but it is included in the maximum likelihood tree with 44% bootstrap support (Figure 1(b)).

There are some lineage-specific subunits of histone-modifying protein complexes. For example, homologues of Dot1, the histone methyltransferase (HMT) catalytic subunit of *Saccharomyces* are present in *K. lactis* and *Y. lipolytica*—2 ascomycetous yeasts (Table 1). Dot1 methylates the histone H3 at lysine-79, which is related to gene silencing in *S. cerevisiae* [14]. This modification system is also found in mammals [14]. However, *S. pombe* has no homologue of Dot1 (Table 1).

**Figure 2:**
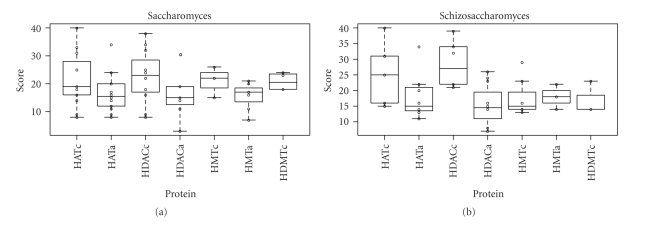
Boxplots of evolutionary conservation levels of subunits of histone-modifying protein complexes. Based on the *E* values of the BLASTP search results, I classified 6 evolutionary conservation levels and scored them as follows: score 0, not detected; score 1, *E* value > 10^0^; score 2, 10^−50^ < *E* value ≤ 10^0^; score 3, 10^−100^ < *E* value ≤ 10^−50^; score 4, 10^−150^ < *E* value ≤ 10^−100^; score 5, *E* value ≤ 10^−150^. Each circle indicates the total of the scores of each protein. Top and bottom boxplots are based on the *Saccharomyces* and *Schizosaccharomyces* proteins, respectively. Boxes are composed of medians with first and third quartiles from the scores of the subunits. HATc, histone acetyltransferase (HAT) catalytic subunits; HATa, HAT-associated subunits; HDACc, histone deacetylase (HDAC) catalytic subunits; HDACa, HDAC-associated subunits; HMTc, histone methyltransferase (HMT) catalytic subunits; HMTa, HMT-associated subunits; and HDMTc, histone demethylase catalytic subunits.

**Figure 3:**
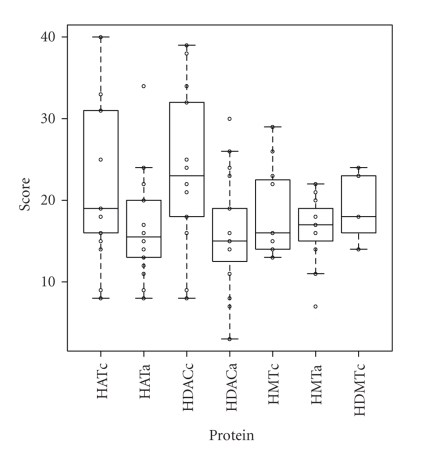
Boxplots of combined evolutionary conservation levels of *Saccharomyces* and *Schizosaccharomyces* subunits of histone-modifying protein complexes. Based on the *E* values of the BLASTP search results, I classified 6 evolutionary conservation levels and scored them as follows: score 0, not detected; score 1, *E* value > 10^0^; score 2, 10^−50^ < *E* value ≤ 10^0^; score 3, 10^−100^ < *E* value ≤ 10^−50^; score 4, 10^−150^ < *E* value ≤ 10^−100^; score 5, *E* value ≤ 10^−150^. Each circle indicates the total of the scores of each protein. Boxes are composed of medians with first and third quartiles from the scores of the subunits. HATc, histone acetyltransferase (HAT) catalytic subunits; HATa, HAT-associated subunits; HDACc, histone deacetylase (HDAC) catalytic subunits; HDACa, HDAC-associated subunits; HMTc, histone methyltransferase (HMT) catalytic subunits; HMTa, HMT-associated subunits; HDMTc, histone demethylase catalytic subunits.

The evolutionary conservation levels of the HAT and histone deacetylase (HDAC) catalytic subunits are higher than those of the associated subunits, respectively, in *Saccharomyces* and *Schizosaccharomyces* (Figure 2). However, the conservation levels of the HMT catalytic subunits are similar to those of the associated subunits, especially in *Schizosaccharomyces* (Figure 2). In order to elucidate the difference in evolutionary conservation levels, I analyzed the combined data of the conservation scores of the HAT catalytic and the associated subunits, HDAC catalytic and the associated subunits, HMT catalytic and the associated subunits, and the histone demethylase (HDMT) catalytic subunits of *Saccharomyces* and *Schizosaccharomyces*. Distributions of the conservation levels of the combined data are shown in Figure 3. The *P* values obtained by the Wilcoxon rank-sum test for the difference between the conservation levels of the HAT catalytic and the associated subunits, HDAC catalytic and the associated subunits, and HMT catalytic and the associated subunits were .043 (<.05), .0027 (<.05), and .90 (>.05), respectively. Thus, the null hypothesis (conservation levels of catalytic and the associated subunits are equal) was rejected in the case of the histone acetylation and deacetylation systems, but not for the histone methylation system.

The results of this study show that histone acetylase and deacetylase catalytic subunits are more conserved than other subunits (Figures 2 and 3). In fact, out of the 10 most abundant proteins of *Saccharomyces* and *Schizosaccharomyces,* 9 were histone acetylation or deacetylation related proteins, that is, 4 HAT catalytic, 3 HDAC catalytic, 1 HAT associated, 1 HDAC associated, and 1 HMT catalytic subunits (Tables 1 and 2). In fungal histone acetylation and deacetylation, the catalytic subunits of protein complexes are conserved and the associated subunits are evolutionary lineage-specific. However, in fungal histone methylation, both the catalytic and the associated subunits are evolutionary lineage-specific. Although the histone modification systems work cooperatively, these results strongly suggest that the evolution of the fungal histone acetylation/deacetylation system was different from that of the histone methylation system.

## Figures and Tables

**Table 1:**
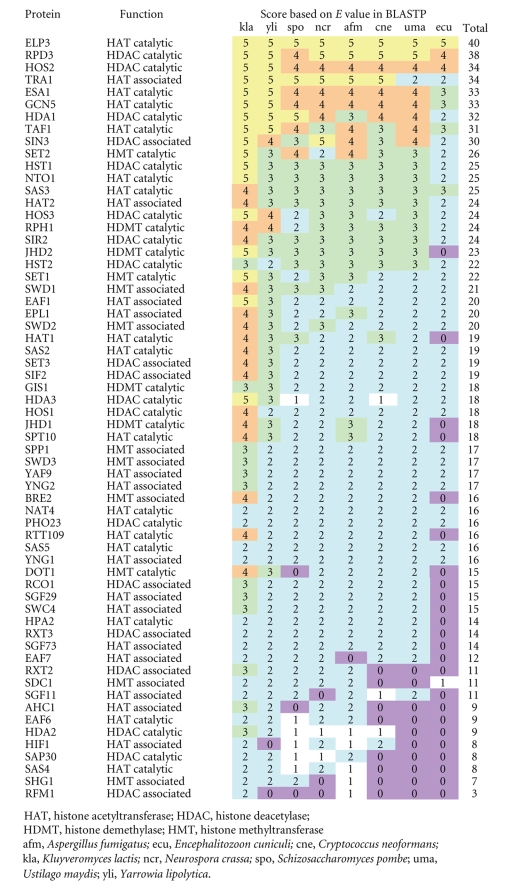
Evolutionary conservation levels of *Saccharomyces* proteins related to histone modifications.

**Table 2:**
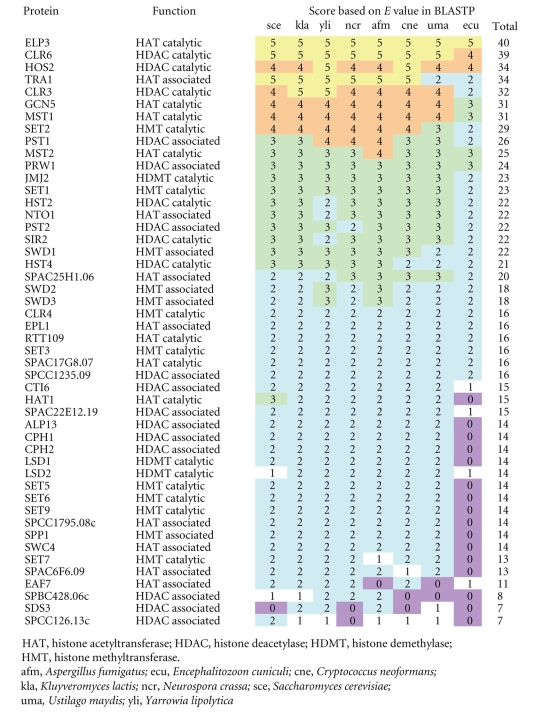
Evolutionary conservation levels of *Schizosaccharomyces* proteins related to histone modifications.
